# Momentary versus retrospective reports of alcohol or cannabis use, sexual activity, and their co-occurrence

**DOI:** 10.1016/j.addbeh.2021.106932

**Published:** 2021-08

**Authors:** Malachi Willis, Tiffany L. Marcantonio, Kristen N. Jozkowski

**Affiliations:** a University of Glasgow, Institute of Health and Wellbeing, MRC/CSO Social and Public Health Sciences Unit, Glasgow, UK; b University of Arkansas, Department of Health, Human Performance, and Recreation, Fayetteville, AR, USA; c Kinsey Institute for Research in Sex, Gender, and Reproduction, Bloomington, IN, USA; d Indiana University, Department of Applied Health Science, Bloomington, IN, USA

**Keywords:** Substance use, Sexual activity, Substance-involved sexual activity, Recall biases, Momentary, Daily diary

## Abstract

**Objective:**

Comparing people’s momentary and retrospective reports of substance use and sexual activity can illuminate discrepant recall biases across these behaviors. Extant research suggests that people tend to underreport alcohol use on retrospective surveys and overreport sexual activity. We provided an updated account of these biases, extending previous work by looking at alcohol- and cannabis-involved sexual activity as well as potential gender differences in recall biases.

**Method:**

Using a sample of adults (*n* = 110; 58.2% women), we administered surveys three times a day for 28 days to measure momentary alcohol and cannabis use, sexual activity, and substance-involved sexual activity. At the end of this momentary assessment, participants completed a retrospective survey assessing how frequently they engaged in these behaviors during the 28-day period.

**Results:**

We compared participants’ momentary reports—which were scaled to account for compliance rates—and retrospective surveys. While there were no significant differences in momentary and retrospective reports of alcohol or cannabis use, participants reported higher rates of sexual activity and alcohol-involved sexual activity on the retrospective surveys than the momentary reports. Effect sizes for significant differences were medium to large (Cohen’s *d*: 0.26–0.67).

**Conclusions:**

Alcohol- and cannabis-involved sexual activity tend to be overreported on retrospective surveys, and preliminary findings suggest that these recall biases may vary by gender. Researchers interested in the co-occurrence of substance use and sexual activity should be aware of this potential random error and consider how to reduce recall biases based on method of data collection.

## 1 Introduction

Substance-involved sexual activity is common (Herbenick et al., 2010), but reported associations between substance use and sexual activity may be affected by people’s ability to accurately remember each of these behaviors. Because evidence suggests that momentary data—which assess behavior shortly after it happens and in natural environments—are more accurate than retrospective reports (Ellison et al., 2020, Graham et al., 2003), researchers can investigate recall biases for behaviors like substance use and sexual activity by comparing reports from retrospective surveys to more proximal assessments from the same participants.

Extant literature indicates that substance use and sexual activity may be differentially affected by recall biases. Specifically, participants tend to underreport drinking alcohol or heavy drinking in retrospective surveys compared with daily surveys (Monk, Heim, Qureshi, Price, & Ryabinin, 2015, Patrick & Lee, 2010), whereas sexual activity is retrospectively overreported (Horvath et al., 2007, Willis and Jozkowski, 2018). Such discrepant recall biases across these behaviors can increase random error and consequently decrease the statistical power of an analysis—making it more difficult for studies using retrospective surveys to identify otherwise significant correlations between substance use and sexual activity (Leigh et al., 1998, Leigh and Stall, 1993).

In addition to distinct methodologies and samples being used to separately assess the recall biases of substance use and sexual activity, a few researchers have investigated these biases for each behavior—as well as their co-occurrence (i.e., substance-involved sexual activity)—within the same study. Similar to sexual activity more generally, alcohol-involved sexual activity may be even more prone to overreporting compared with sexual behavior that was not preceded by alcohol use (Graham et al., 2003, Leigh et al., 1998). Although both of these studies assessed “other” substance use, neither reported findings on specific substances other than alcohol.

Using data from a study designed to assess within-person variability of sexual consent (Willis, Jozkowski, Bridges, Veilleux, & Davis, 2021), the purpose of this short communication was three-fold. First, we provided an updated account regarding whether the recall biases for alcohol use, sexual activity, and the co-occurrence of these behaviors are discrepant. Second, we contributed preliminary data on potential recall biases for cannabis-involved sexual activity. Finally, we examined whether recall biases for alcohol- or cannabis-involved sexual activity varied by gender—an individual difference that may be relevant to recall biases for alcohol use (Patrick & Lee, 2010) but not sexual activity (Gillmore, Leigh, Hoppe, & Morrison, 2010).

## 2 Method

### 2.1 Participants

Participants were recruited via social media (e.g., study recruitment pages on Reddit and Facebook) and a campus-wide e-newsletter at a university in the southern United States. To be eligible, participants had to be at least 18 years old, have daily access to a smartphone, and be sexually active. Of the 545 people who completed the screener survey, we invited 40.0% to participate in the 28-day study. Of these, 72.9% completed the baseline survey; however, 7.5% of those participants never downloaded the momentary survey application onto their personal devices. In sum, 138 people began this 28-day study; 18.1% withdrew from the study for personal or unknown reasons, and 5.1% did not complete all items of interest on the surveys. Thus, the final analytic sample comprised 110 participants.

On average, the participants included in the analytic sample were 29.4 years old (*SD* = 6.6), ranging from 21 to 65. About half (47.3%) were graduate students, 11.8% were undergraduate students, and 40.9% were not students. Regarding gender, 58.2% identified as women, 40.9% as men, and 0.9% as gender fluid. For sexual orientation, 75.5% identified as heterosexual, 15.5% as bisexual, and 9.1% as another orientation. All participants reported being in a committed relationship with their current sexual partner, ranging from 0.2 to 35.3 years in length (*M* = 5.9, *SD* = 5.9). Most participants (89.1%) were in the United States at the time of the study; 70.0% identified as White/European American, 11.8% as Hispanic/Latin American, 10.0% as Asian/Asian American, 5.5% as another race/ethnicity, and 2.7% as multiple races/ethnicities.

### 2.2 Procedure

Sociodemographic variables were measured during a baseline survey via Qualtrics. To assess momentary behavior, surveys were sent to participants via LifeData three times a day for 28 days using a semi-random sampling scheme: 7am–9am, 1pm–3pm, and 7pm–9pm (83.8% completion rate). To assess retrospective behavior, participants completed an exit survey via Qualtrics within two days of the 28-day study (95.7% completion rate). On average, participants completed momentary surveys on 26.7 of the 28 days (*SD* = 2.7), ranging from 15 to 28. Participants received up to a $40 USD e-gift card for their participation. The procedure for this study was approved by the university’s institutional review board.

### 2.3 Measures

#### 2.3.1 Momentary behavior

In the momentary surveys, participants reported their recent substance use. If participants had engaged in sexual activity since the previous survey, they recorded their substance use before or during the sexual event. Otherwise, they reported substance use since the last survey prompt. Response options for alcohol were presented on a 7-point sliding scale: 0 to 6+. To determine whether participants had consumed at least five alcoholic drinks on a given day (i.e., heavy drinking), we created sum scores across each day’s three momentary surveys. Response options for cannabis use were dichotomous (0 = no; 1 = yes). Measuring alcohol consumption and heavy drinking by number of drinks over the course of a day and cannabis use dichotomously is consistent with previous daily diary research (e.g., Shorey, Moore, McNulty, & Stuart, 2016).

Regarding sexual activity, participants indicated whether they had engaged in partnered sexual activity and checked all that applied from a list of sexual behaviors, which is consistent with similar research (e.g., Willis & Jozkowski, 2018).

#### 2.3.2 Retrospective behavior

In the exit survey, we asked participants to reflect on the 28-day study and report on how many days they engaged in alcohol use, cannabis use, sexual activity, and alcohol- or cannabis-involved sexual activity. For each behavior, participants selected from a dropdown menu that ranged from “0 days” to “28 days.”

### 2.4 Analysis

To compare the data reported on the momentary and retrospective surveys, we conducted paired-samples *t*-tests. For each comparison, we excluded participants who did not report a single instance of a behavior on the momentary surveys. We also scaled the momentary data to account for the number of days that participants responded to the survey. To assess gender differences regarding proportions of participants who over-/underreported behaviors on the retrospective surveys compared with the momentary surveys, we conducted chi-squared tests of independence.

For the paired-samples *t*-tests, we assessed the assumption that mean differences should be normally distributed (i.e., skewness < 2; kurtosis < 7). If a mean difference did not meet this assumption, we provided robust estimates of the *t*-statistic and *p*-value that were derived based on 1000 bootstrap samples. To control for inflated Type 1 error rates due to multiple tests and maintain a familywise alpha of 0.05, we adjusted the reported *p*-values separately for the 13 *t*-tests and 13 χ^2^-tests according to the Benjamini and Hochberg (1995) procedure for controlling the false discovery rate. Complementing tests of significance, we reported effect sizes (i.e., Cohen’s *d* and Cramer’s *V*). All analyses were conducted using SPSS 27.

## 3 Results

Across all person-days (*n* = 3080), at least one alcoholic drink was consumed on 906 days (29.4%), at least five alcoholic drinks were consumed on 98 days (3.2%), and cannabis was used on 266 days (8.6%). Momentary and retrospective reports were strongly correlated for each type of substance use: consuming at least one alcoholic drink, *r* = 0.72, *p* < .001, consuming at least five alcoholic drinks, *r* = 0.45, *p* = .018, and using cannabis, *r* = 0.80, *p* < .001. There were not significant differences between momentary and retrospective reports for these behaviors, and effects sizes were negligible (Table 1). As such, the proportion of participants who over- or underreported alcohol or cannabis use on the retrospective versus momentary surveys were similar; these proportions did not vary by gender (Table 2).

**Table 1:** Number of Days Participants Reported Engaging in Substance Use and Sexual Activity on Momentary and Retrospective Surveys.

		Momentary Reports				Momentary Reports (Scaled)				Retrospective Reports				*t* tests^1^			
	*n*	*M*	*SD*	Range		*M*	*SD*	Range		*M*	*SD*	Range		*t*	*df*	*p*	*d*
**Substance use**																	
Have ≥ 1 drink	90	10.07	7.32	1–25		10.44	7.43	1–25		10.53	8.32	0–28		−0.15	89	0.877	0.016
Have ≥ 5 drink	27	3.71	3.38	1–19		3.71	4.96	1–19.70		2.70	3.38	0–15		1.15	26	0.261	0.221
Use cannabis	33	8.06	7.49	1–27		8.51	7.74	1–27		8.39	9.23	0–28		0.12	32	0.907	0.020

**Sexual activity**																	
Passionate kissing	105	7.44	5.05	1–22		7.79	5.27	1–26.73		11.92	7.71	1–28		−6.75***	104	<0.001	0.658
Genital touching (give)	105	6.71	4.31	1–21		7.07	4.55	1–26.73		8.68	6.09	1–28		−3.03**	104	0.003	0.296
Genital touching (receive)	106	6.57	4.09	1–20		6.91	4.33	1–25.45		8.80	6.06	1–28		−4.54***	105	<0.001	0.441
Oral sex (give)	81	4.62	4.03	1–19		4.85	4.32	1–24.18		5.84	5.14	0–23		−2.36*	80	0.020	0.263
Oral sex (receive)	78	4.28	2.79	1–18		4.51	4.18	1–22.91		5.42	4.78	0–23		−3.14**	77	0.002	0.355
Vaginal sex	101	6.83	4.09	1–20		7.23	4.43	1–25.45		8.59	5.72	1–26		−3.84***	100	<0.001	0.382
Anal sex	12	3.33	3.65	1–12		3.98	4.69	1–15.27		6.50	7.13	0–20		−2.33†	11	0.040	0.674

**Substance-involved sex. act.**																	
Sex. act. with ≥ 1 drink	90	2.19	2.49	0–11		2.25	2.55	0–11.41		3.38	4.05	0–20		−3.18**	89	0.002	0.336
Sex. act. with ≥ 5 drink	27	0.70	1.49	0–5		0.73	1.54	0–5.19		1.26	3.38	0–8		−1.82*	26	0.080	0.350
Sex. act. with cannabis^2^	33	2.33	2.20	0–8		2.44	2.26	0–8.30		4.09	6.54	0–27		−1.75	32	0.128	0.296

*Note.*^1^Paired samples *t*-tests comparing the scaled momentary reports with the retrospective reports.

^2^This mean difference was not normally distributed; the reported *t*-statistic and corresponding *p*-value are robust estimates derived based on 1000 bootstrap samples.

*p < .05. **p < .01. ***p < .001. †This *t*-value was no longer significant (α = 0.05) once adjusting the *p*-value according to the Benjamini and Hochberg (1995) procedure for controlling the false discovery rate.

**Table 2:** Percentage of Participants Who Over-/Underreported Engaging in Substance Use and Sexual Activity on Retrospective versus Momentary Surveys.

	Women				Men				χ^2^ tests			
	*n*	Over (%)	Same (%)	Under (%)	*n*	Over (%)	Same (%)	Under (%)	χ^2^	*df*	*p*	φ_C_

**Substance use**												
Have ≥ 1 drink	55	45.5	18.2	36.4	34	47.1	17.6	35.3	0.02	2	0.989	0.016
Have ≥ 5 drink	13	30.8	15.4	53.8	14	42.9	7.1	50.0	0.70	2	0.706	0.161
Use cannabis	14	50.0	7.1	42.9	19	31.6	15.8	52.6	1.35	2	0.509	0.202

**Sexual activity**												
Passionate kissing	63	74.6	6.3	19.0	41	75.6	7.3	17.1	0.09	2	0.956	0.030
Genital touching (give)	63	58.7	6.3	34.9	41	63.4	4.9	31.7	0.26	2	0.878	0.050
Genital touching (receive)	62	61.3	16.1	22.6	43	72.1	7.0	20.9	2.20	2	0.333	0.145
Oral sex (give)	51	47.1	27.5	25.5	29	62.1	13.8	24.1	2.34	2	0.310	0.171
Oral sex (receive)	43	53.5	25.6	20.9	34	55.9	23.5	20.6	0.05	2	0.974	0.026
Vaginal sex	61	55.7	19.7	24.6	40	60.0	15.0	25.0	0.37	2	0.829	0.061
Anal sex	5	40.0	20.0	20.0	7	71.4	14.3	14.3	1.32	2	0.516	0.332

**Substance-involved sex. act.**												
Sex. act. with ≥ 1 drink	55	61.8	27.3	10.9	34	32.4	41.2	26.5	7.87†	2	0.020	0.297
Sex. act. with ≥ 5 drink	13	46.2	38.5	15.4	14	42.9	57.1	0.0	2.66	2	0.265	0.314
Sex. act. with cannabis	14	50.0	21.4	28.6	19	36.8	42.1	21.1	1.55	2	0.461	0.217

*Note.* †This χ^2^-value was no longer significant (α = 0.05) once adjusting the *p*-value according to the Benjamini and Hochberg (1995) procedure for controlling the false discovery rate.

Momentary and retrospective reports were also strongly correlated for all sexual behaviors, *r*s = 0.51–84, *p*s < 0.001. However, significantly more instances of each sexual behavior were reported on the retrospective survey than on the momentary surveys—with medium to large effect sizes (Table 1). Demonstrating these discrepancies, proportionally more participants retrospectively overreported engaging in each of the sexual behaviors; again, there were not gender differences (Table 2).

Across all person-days with sexual behavior (*n* = 978), at least one alcoholic drink was consumed before engaging in partnered sexual activity on 197 days (20.1%), at least five alcoholic drinks were consumed beforehand on 19 days (1.9%), and cannabis was used beforehand on 77 days (7.9%). Momentary and retrospective reports were strongly correlated for sexual activity that involved each type of substance use: at least one alcoholic drink, *r* = 0.56, *p* < .001, at least five alcoholic drinks, *r* = 0.62, *p* < .001, and cannabis, *r* = 0.56, *p* < .001. However, participants tended to report higher rates of sexual activity that involved at least one alcoholic drink on the retrospective survey than on the momentary surveys (Table 1). Further, proportionally more women (61.8%) than men (32.4%) overreported their alcohol-involved sexual activity on the retrospective survey (Table 2).

## 4 Discussion

Our finding that sexual activity is more prone to being overreported on retrospective surveys than alcohol or cannabis use corroborates previous studies. Because discrepant measurement biases across constructs can affect estimates of their association (Leigh et al., 1998, Leigh and Stall, 1993), researchers interested in studying substance use and sexual activity should consider collecting momentary data when resources are available or aim to reduce recall biases in other ways if using retrospective surveys (e.g., using context-specific question formats; McAuliffe, DiFranceisco, & Reed, 2007).

However, we did not find that momentary reports of alcohol and cannabis use varied from their retrospective surveys—inconsistent with prior work that has suggested people retrospectively underreport their substance use. Potentially explaining this, we asked participants to retrospectively report number of days rather than the more specific assessment (i.e., number of drinks per day) used in other studies (Monk, Heim, Qureshi, Price, & Ryabinin, 2015, Patrick & Lee, 2010). An alternative explanation is that recall biases were systematically underestimated because participants were asked to retrospectively report their substance use behavior for the same period that they provided momentary data (Poulton, Pan, Bruns, Sinnott, & Hester, 2018); future work could address this concern by using the timeline follow-back procedure (TLFB; Sobell & Sobell, 1992).

We also found that alcohol- and cannabis-involved sexual activity tended to be overreported on the retrospective survey. While the recall bias for cannabis-involved sexual activity was of the same direction and similar magnitude as alcohol-involved sexual activity, this difference was not statistically significant—likely due to cannabis use being less prevalent in this sample. Neither Nalcohol- nor cannabis-involved sexual activity was more prone to overreporting than general sexual activity. Although researchers have suggested that recall biases may be greater for substance-involved sexual activity (Leigh et al., 1998), the effect of concurrent alcohol use on recall bias for vaginal sex is weak and diminishes when accounting for relevant variables like retrospective survey time lag or orgasm experience (Graham et al., 2003).

Finally, we provided preliminary evidence that recall biases for substance-involved sexual activity might vary by gender. Specifically, women may be more likely than men to overreport their alcohol-involved sexual activity, which aligns with evidence that this trend occurs with recall biases for alcohol use more generally (Patrick & Lee, 2010). Further work is needed to better understand the effects of gender and other individual differences on recall biases for substance-involved sexual activity.

A primary limitation of our study is that all data presented were based on self-reports, which may be subject to social desirability biases. And even though our sample size was much larger than typical experience sampling methodology studies (van Berkel, Ferreira, & Kostakos, 2017), our analyses regarding behaviors that occurred less frequently (e.g., cannabis-involved sexual activity) were underpowered. Further, the generalizability of our findings is limited because the sample primarily comprised White heterosexual participants who were in a committed relationship at the time of the study. Finally, pandemic-related social restrictions social may have influenced people’s substance use or sexual activity; however, we systematically controlled for this potential confounding factor by collecting all data from the same period.

Understanding and addressing discrepant recall biases across alcohol or cannabis use and sexual activity is critical for obtaining valid conclusions, which may ultimately inform interventions for public health problems like substance-facilitated sexual assault (Abbey, 2002, Shorey et al., 2016) or inconsistent condom use when under the influence of substances (Kiene et al., 2009, Abbey et al., 2007).

## Role of funding sources

This research was funded in part by student research grants awarded by the Graduate-Professional Student Congress and Department of Health, Human Performance, and Recreation at the University of Arkansas. This work was also supported by the United Kingdom Medical Research Council (MC_UU_00022/3) and the Scottish Government Chief Scientist Office (SPHSU18). These funders had no role in the study design, collection, analysis or interpretation of the data, writing the manuscript, or the decision to submit the paper for publication.

## Contributors

Malachi Willis and Kristen N. Jozkowski designed the study and wrote the protocol. Malachi Willis and Tiffany L. Marcantonio conducted literature searches. Malachi Willis conducted the statistical analysis and wrote the first draft of the manuscript. All authors contributed to and have approved the final manuscript.

## CRediT authorship contribution statement

**Malachi Willis:** Conceptualization, Methodology, Formal analysis, Investigation, Investigation, Funding acquisition, Data curation, Writing - original draft, Project administration, Funding acquisition. **Tiffany L. Marcantonio:** Writing - review & editing. **Kristen N. Jozkowski:** Conceptualization, Writing - review & editing, Supervision.

## Declaration of Competing Interest

The authors declare that they have no known competing financial interests or personal relationships that could have appeared to influence the work reported in this paper.
